# The relationships between empathy, stress and social support among medical students

**DOI:** 10.5116/ijme.55e6.0d44

**Published:** 2015-09-05

**Authors:** Kyung Hye Park, Dong-hee Kim, Seok Kyoung Kim, Young Hoon Yi, Jae Hoon Jeong, Jiun Chae, Jiyeon Hwang, HyeRin Roh

**Affiliations:** 1 Department of Emergency Medicine, Inje University College of Medicine, Busan, Republic of Korea; 2 Inje University College of Medicine, Busan, Republic of Korea; 3 Department of Medical Education, Inje University College of Medicine, Busan, Republic of Korea

**Keywords:** Medical student, empathy, social support, stress

## Abstract

**Objectives:**

To examine the relationship between stress, social support, and empathy among medical students.

**Methods:**

We evaluated the relationships between stress and empathy, and social support and empathy among medical students. The respondents completed a question-naire including demographic information, the Jefferson Scale of Empathy, the Perceived Stress Scale, and the Multidimensional Scale of Perceived Social Support. Corre-lation and linear regression analyses were conducted, along with sub-analyses according to gender, admission system, and study year.

**Results:**

In total, 2,692 questionnaires were analysed. Empathy and social support positively correlated, and empathy and stress negatively correlated. Similar correla-tion patterns were detected in the sub-analyses; the correla-tion between empathy and stress among female students was negligible. In the regression model, stress and social support predicted empathy among all the samples. In the sub-analysis, stress was not a significant predictor among female and first-year students.

**Conclusions:**

Stress and social support were significant predictors of empathy among all the students. Medical educators should provide means to foster resilience against stress or stress alleviation, and to ameliorate social support, so as to increase or maintain empathy in the long term. Furthermore, stress management should be emphasised, particularly among female and first-year students.

## Introduction

Empathy in medical practice is related to clinical competence.^1^ Low levels of empathy lead to patient dissatisfaction, problems in medical communication, and even medical errors.^1^^,^^2^ The assumed reasons for low empathy levels among senior medical students are the lack of good role models, a high academic workload, and time pressure.^3^^,^^4^ Demographic factors, including gender, the medical school admission system (undergraduate programme, post-baccalaureate programme), and study-year level, are also some of the reasons.^4^ In addition, burnout or a negative mood state play an integral role in the level of empathy among medical students and interns.^5^^-^^8^ More than 80% of medical students have at least one form of distress, and more than half have had more than three forms of distress including burnout, low quality of life, depression, sleepiness, and stress.^9^ Stress among medical students leads to alcohol and substance use or, in extreme cases, suicide.^9^^,^^10^ High levels of stress tend to continue when medical students become physicians.^11^

Social support is one of the coping mechanisms for stress. Medical students satisfied with their own lives have been found to have low perceived stress and high resilience, and to seek social support.^12^ In addition, social support and good relationships with family members and friends increase quality of life.^13^ Low social support is related to poor academic self-perception, as well as mental health problems among medical students.^14^^-^^17^ Like stress, insufficient social support might continue after these students’ qualification as doctors, which could affect their medical practice. Low social support may induce low levels of empathy, as low social support is closely associated with stress and, in turn, stress is associated with low empathy levels. However, there is little research on the association between empathy and social support among medical students.

We assume that high stress levels and low social support are some of the important contributing factors towards low empathy levels among medical students. However, little is known about the relationships between stress, social support, and empathy, particularly as determined through use of the Jefferson Scale of Empathy (JSE). Most previous studies on stress and empathy did not use the JSE, an instrument designed to measure empathy in relation to a patient-caregiver situation.^1^ We evaluated the relationship between stress and empathy, and between social support and empathy among medical students. In addition, to analyse the nature of these relationships according to gender, admission system, and study-year level, and to ensure that tailored suggestions for the students would be made, sub-analyses were conducted.

## Methods

### Participants

This cross-sectional study was approved by the institutional review board for human research at Inje University Busan Paik Hospital, South Korea. The questionnaires were distributed to all first- to fourth-year medical students at 20 medical schools in South Korea, in October 2014. The following data were also collected, forming part of demographic data: gender, age, medical school admission systems, and study-year level.

### Instruments

The student version of the JSE was used. The JSE was developed so as to measure physician empathy,^18^ and was translated into Korean in 2010, yielding a Cronbach’s alpha of 0.84.^19^ The JSE has three underlying constructs of empathy, comprising 10 items on perspective taking (PT), 8 on compassionate care (CC), and 2 on standing in the patient’s shoes (SP). Each item is rated on a 7-point Likert scale. The total score is the sum of all item scores. A higher score indicates a higher degree of empathy (maximum score = 140).

The Perceived Stress Scale (PSS) is as self-report instrument used to evaluate the degree of perceived stress in daily life within the past month. It was developed by Cohen et al. in 1983, who at the time, reported good reliability and validity.^20^ Lee et al. translated the PSS into Korean, and reported a Cronbach’s alpha of 0.82, which indicated good reliability and validity.^21^ The PSS is a 10-item self-administered questionnaire and each item is rated on a 5-point Likert scale. The total score is the sum of all item scores. A higher score is indicative of greater perceived stress (maximum score = 40).

The Multidimensional Scale of Perceived Social Support (MSPSS) was developed by Zimet *et al *^22^ the scale measures respondents’ perceived support from three sources, namely, family, friends, and significant others. It was translated into Korean and yielded good reliability and validity.^23^ The MSPSS is a brief instrument consisting of 12 items; each item is rated on a 7-point Likert scale. The total score is the sum of all item scores. A higher score indicates a higher level of perceived social support (maximum = 84).

### Statistical analyses

The differences in the levels of empathy, social support, and stress according to gender and medical education system were evaluated using the t-test, whereas analysis of variance (ANOVA) was used to analyse those differences according to study-year level, with Dunnett’s T3 test used for post-hoc analysis. Pearson’s correlation analysis was performed to assess the relationship between empathy and social support, and between empathy and stress. Multiple linear regression analysis (enter method) was used to identify predictors of empathy. The Statistical Package for the Social Sciences (ver. 20.0, SPSS Inc., Chicago, IL, USA) was used for the analyses, and significance was declared at p < 0.05.

## Results

In total, 2,702 questionnaires were received from 20 medical schools. Questionnaires with incomplete data were excluded and, consequently, 2,692 questionnaires were used for analysis. The sample comprised 1,675 male (62.2%) and 1,017 female students (37.8%). The mean age of the respondents was 24.73 years (range: 20–40).  In addition, 1,353 students were enrolled in undergraduate programmes (50.3%) and 1,339 enrolled in post-baccalaureate programmes (49.7%). There were 929 (34.5%) first-year students, 971 (36.1%) second-year students, 559 (20.8%) third-year students, and 233 (8.7%) fourth-year students (Table 1).

**Table 1:** Respondents’ demographic characteristics

Variables		No. (%)
Gender		
	Male	1,675 (62)
	Female	1,017 (38)
Age		
	Years (Mean, SD)	24.73 (3.004)
Medical school admission system		
	Undergraduate programme	1,353 (50)
	Post-baccalaureate programme	1,339 (50)
Medical school admission system and sex		
	Males in undergraduate programme	912 (67)
	Females in undergraduate programme	441 (33)
	Males in post-baccalaureate programme	763 (57)
	Females in post-baccalaureate programme	576 (43)
Study year		
	First-year	929 (35)
	Second-year	971 (36)
	Third-year	559 (21)
	Fourth-year	233 (9)
Total		2,692 (100)

### Jefferson Scale of Empathy

The mean total score on the JSE was 105.48 ± 14.67 (Table 2) and the Cronbach’s alpha value obtained for the JSE was 0.715. The mean total score obtained by female students (106.63 ± 13.25) on the JSE was higher than that of male students (104.76 ± 15.44) (p=0.001) (Table 2). Students enrolled in the post-baccalaureate programme (106.17 ± 14.46) had a higher mean total JSE score than did students in the undergraduate programme (104.77 ± 14.86) (p= 0.014) (Table 2). The mean total JSE scores obtained by students in the different study-year levels ranked in the following descending order: first, third, fourth, and second-year (p=0.005). Post-hoc comparisons showed that the second-year students obtained lower scores than did first-year students (p=0.003).

**Table 2:** The JSE, PSS, and MSPSS according to variables

Variables		JSE^a^Mean (SD)	PSS^b^Mean (SD)	MSPSS^c^Mean (SD)
Total students		105.47(14.67)	28.65(6.22)	68.02(11.79)
Gender				
	Male	104.76(15.44)	28.26(6.25)	68.34(12.26)
	Female	106.63(13.25)*	29.29(6.12)*	70.14(10.90)*
Medical school admission system				
	Undergraduate programme	104.77(14.86)	28.85(6.28)	67.72(12.40)
	Post-baccalaureate programme	106.17(14.46)†	28.45(6.15)	70.35(10.99)†
Medical school admission system and sex				
	Males in undergraduate programme	103.58(15.36)	28.54(6.22)	66.89(12.70)
	Females in undergraduate programme	107.24(13.44)*	29.49(6.37)*	69.42(11.58)*
	Males in post-baccalaureate programme	106.17(15.42)	27.93(6.28)	70.08(11.47)
	Females in post-baccalaureate programme	106.16(13.09)	29.14(5.92)*	70.69(10.32)
Study years				
	First-year	106.80(13.92)	29.55(6.11)	69.66(11.07)
	Second-year	104.46(15.52)ǂ	28.60(6.05)ǂ	68.15(12.61)ǂ
	Third-year	105.32(14.49)	27.75(6.58)	69.37(11.84)ǂ
	Fourth-year	104.70(14.12)	27.47(5.96)	69.25(10.72)ǂ

^a^JSE: Jefferson Scale of Empathy; ^b^PSS: Perceived Stress Scale; ^c^MSPSS: Multidimensional Scale of Perceived Social Support.*p<0.05 when compared to male participants; †p<0.05 when compared to the undergraduate programme; ǂp<0.05 when compared to first-year level

### Perceived stress scale

The mean total score on the PSS was 28.65 ± 6.22 (Table 2) and the Cronbach’s alpha value obtained for the PSS was 0.638. The mean total PSS score obtained by female students (29.29 ± 6.12) was higher than that of male students (28.26 ± 6.25) (p<0.001) (Table 2). There were no differences in the mean total PSS scores obtained, according to medical school admission systems (p=0.093) (Table 2). The mean total PSS scores obtained by students in the different study-year levels ranked in the following descending order: first-, second-, third-, and fourth-year (Table 2). This indicates that the students in the higher study-year levels seemingly obtained lower stress scores. Post-hoc comparisons showed that first-year students obtained higher scores than did second, third, and fourth-year students (p= 0.004, p < 0.001, p < 0.001, respectively).

### Multidimensional scale of perceived social support

The mean total score on the MSPSS was 68.02 ± 11.79 (Table 2) and the Cronbach’s alpha value obtained for the MSPSS was 0.930. The mean total MSPSS score obtained by female students (70.14 ± 10.90) was also higher than that of male students (68.34 ±12.26) (p< 0.001) (Table 2). Students enrolled in the post-baccalaureate programme (70.35 ± 10.99) obtained a higher mean total MSPSS score than did their counterparts (67.72 ± 12.40) (p< 0.001) (Table 2). The mean total MSPSS scores obtained by students in the different study-year levels ranked in the following descending order: first, third, fourth, and second-year. Post-hoc comparisons revealed that first-year students obtained higher scores than did second-year students (p=0.033) (Table 2).

### Correlation between empathy, stress, and social support

The JSE score and the PSS score showed a weak, negative correlation (r=-0.14, p<0.001). In the sub-analyses comprising the medical school admission system and study-year level, correlations between empathy and stress were similar to those obtained for all of the students. However, a negligible correlation between empathy and stress was detected among female students (r = -0.07, p = 0.021) (Table 3).

The JSE score and the MSPSS score showed a moderate, positive correlation (r = 0.33, p < 0.001). In the sub-analyses comprising gender, the medical school admission system, and study-year level, correlations between empathy and social support were similar to those obtained by all of the students (Table 3).

**Table 3:** Pearson’s correlation coefficients obtained for the JSE, PSS, and the MSPSS

Variables		JSE^a^ and PSS^b^	JSE^a^ and MSPSS^c^
Total students		-0.14*	0.33*
Gender			
	Male	-0.19*	0.34*
	Female	-0.07†	0.29*
Medical school admission system			
	Undergraduate programme	-0.14*	0.32*
	Post-baccalaureate programme	-0.15*	0.32*
Study year			
	First-year	-0.13*	0.33*
	Second-year	-0.14*	0.35*
	Third-year	-0.18*	0.29*
	Fourth-year	-0.21†	0.25*

^a^JSE: Jefferson Scale of Empathy; ^b^PSS: Perceived Stress Scale; ^c^MSPSS: Multidimensional Scale of Perceived Social Support.*p<0.001, †p<0.05

### Regression analysis of stress and social support

In the regression model, stress and social support were found to be moderate, significant predictors of empathy among all of the students. Social support and stress showed similar patterns of moderate, significant prediction of empathy in the sub-analysis. However, in the sub-analysis, stress did not significantly predict empathy among female and first-year students, respectively (Table 4).

**Table 4:** Stress and social support as predictors of empathy

Variable	PSS^a^			MSPSS^b^		
	B	SE	β	B	SE	β
Total students (R^2^ = 0.109)	-0.144*	0.044	-0.061	0.385†	0.023	0.309
Gender						
Male (R^2^ = 0.122)	-0.246†	0.059	-0.100	0.387†	0.030	0.308
Female (R^2^ = 0.086)	-0.002	0.067	-0.001	0.356†	0.038	0.293
Admission system						
Undergraduate programme (R^2^ = 0.108)	-0.154ǂ	0.062	-0.065	0.368†	0.032	0.307
Post-baccalaureate programme (R^2^ = 0.107)	-0.129ǂ	0.064	-0.055	0.404†	0.036	0.307
Study year						
First-year (R^2^ = 0.108)	-0.106	0.073	-0.046	0.398†	0.040	0.316
Second-year (R^2^ = 0.127)	-0.166ǂ	0.079	-0.065	0.415†	0.038	0.337
Third-year (R^2^ = 0.090)	-0.198ǂ	0.095	-0.090	0.313†	0.053	0.256
Fourth-year (R^2^ = 0.081)	-0.349ǂ	0.156	-0.147	0.269*	0.087	0.205

B: unstandardized coefficient; SE: standard error; β: standardized coefficient. ^a^PSS: Perceived Stress Scale;^b^MSPSS: Multidimensional Scale of Perceived Social Support.*p<0.01, †p<0.001; ǂp<0.05

## Discussion

In our study, we demonstrated the association between empathy and stress, and between empathy and social support in a large sample of Korean medical students. However, we found a negligible correlation between empathy and stress among female students and no significant prediction of empathy by stress among female students and first-year students, respectively.

### Empathy and stress

The mean total score on the PSS in our study was surprisingly higher than that previously obtained by medical students in the United States (16.6 ± 7.49) and Chinese university students (18.44 ± 7.35).^9^^,^^24^ Particularly, in our study, female students enrolled in the two medical school admission systems showed high levels of stress, consistent with previous studies.^7^^,^^11^ Female students showed personal distress, emotional exhaustion, and low perceived physical and mental quality of life than did males in the previous studies.^7^^,^^11^ Female physicians have also shown higher stress prevalence and less resistance to stress than have their male countparts.^14^^,^^25^ Compared to their female counterparts, male internal medicine residents have displayed higher levels of mental well-being.^26^ Moreover, female medical students showed higher levels of empathy and stress levels, respectively, as compared to male students. Women’s sensitivity to emotional states might correlate with high empathy and stress levels among female medical students. This might mean that female students are more sensitive to stress, even though their stress levels are similar to those of males.

In this study, first-year medical students showed the highest stress levels; a decline in stress levels among students in the higher study-year levels was also observed. In a previous longitudinal study, first-year students had the highest levels of depression and anxiety, contrary to fourth-year students, who had the lowest depression and anxiety levels.^27^ This trend could be attributed to better adjustment by senior students to stressful circumstances and to the decline in the burden of the high frequency of examinations taken by this group of students. Certainly, freshmen are confronted with a considerable amount of distress, as they are newly exposed to a competitive and hierarchical culture and are aware of expectations by their families and society.

In our study, a negative correlation was found between scores on stress and empathy levels. This finding is consistent with previous studies, wherein empathy negatively correlated with burnout among medical students.^6^^-^^8^ In another study, negative mood states (e.g. distress, depression, anger, and fatigue) increased significantly during the internship; these correlated negatively with empathic concern.^5^ Stress, depression, and burnout among physicians also reduce empathy, which leads to sub-optimal patient care practice and attitudes, sometimes even medical errors.^2^^,^^28^^-^^30^ Stress results in depersonalization and emotional exhaustion, which might explain the correlation between stress and empathy.

In the regression analysis, stress did not correlate significantly with empathy among female and first-year students, respectively. This might be because women are biologically emotional, have high levels of empathy, and are sensitive to stress, as previously mentioned. Furthermore, first-year students might experience high levels of stress due to excessive competition and a new environment, though retaining sufficient levels of empathy. Senior students seem to become insensitive to stress and less empathetic, due to depersonalization.

### Empathy and social support

In our study, the total mean score on the MSPSS was 68.02 ± 11.79, which is similar to the score previously obtained by Chinese university students (69.12 ± 10.14).^24^ Moreover, female students had higher social support than did male students, similar to a previous study on female physicians.^25^ Male students and undergraduate students had lower levels of social support than did their respective counterparts (Table 2). In addition, the mean score on the social support scale was lowest among male students enrolled in an undergraduate programme (Table 2). The majority of undergraduate students enrolled in medical school immediately after graduating from high school, therefore, easily becoming socially isolated once in medical school. In addition, unlike female students, male students might have difficulties establishing intimate relationships.

Weak social support, measured through the Medical Outcome Study, is commonly associated with mental problems among medical students.^16^ In a study, medical students with no social support had a higher prevalence of depression than did those who could get help from family and/or friends.^14^ In our study, social support positively correlated with empathy; this was demonstrated in the regression analysis and all the sub-analyses. In the general adult population, empathy is also closely associated with social interaction.^31^ Good social support can buffer against psychological stress, which helps maintain empathy among medical students, too.

We have made several suggestions for medical education on the basis of the above results. It is almost impossible to remove or reduce all the stressors affecting medical students. However, medical students should be provided with tools that aid the development of resilience against stress and the means to modify or decrease the impact of stressors, as part of the curriculum. For example, students with problem-based learning felt more socially supported at university than did those on the traditional curriculum tracks.^32^ As mentioned before, professional stress continues after students’ graduation from medical school and qualification as doctors. Therefore, medical students should be taught how to manage stress and seek social support. This would lead to the maintenance of or an increase in empathy after their qualification as physicians. Lastly, medical educators should consider gender differences when delivering educational programmes on professionalism, as Paro *et al* have suggested.^7^

### Limitations

The present study has a few limitations. The response rate was 48%, due to the low response rate of third- and fourth-year students. There was limited access to third- and fourth-year students during the course of our survey. Third-year students were conducting clinical work and fourth-year students were preparing for the medical licensing examination. Furthermore, in addition to stress and social support, various other factors may be influencing empathy among medical students. This limitation could be the reason for the low correlation coefficient or weak prediction yielded by the regression analysis. Finally, our result regarding stress might be temporary, as PSS is used to measure stress levels relating to the previous month. However, our study might be representative of the typical Korean medical student, as it included multiple schools and students in various medical fields.

## Conclusions

Stress and social support were significant predictors of empathy among all the students. In the sub-analyses conducted according to gender, the medical school admission system, and study-year level, similar patterns were demonstrated; however, stress was not a significant predictor among female students and first-year students. Medical educators should construct measures aimed at developing resilience against stress or decreasing stress, and at improving social support; examples of these are the restructuring of the curriculum and the mental health support system. These would lead to the maintenance of or an increase in empathy in the long term. Furthermore, stress management should be emphasised, particularly among female and first-year students.

### Acknowledgement

This work was supported by the Inje University College of Medicine, in 2014.

### Conflict of interest

The authors declare that they have no conflict of interest.

## References

[1] Hojat M, Gonnella JS, Mangione S, Nasca TJ, Veloski JJ, Erdmann JB, Callahan CA, Magee M (2002). Empathy in medical students as related to academic performance, clinical competence and gender.. Med Educ.

[2] West CP, Huschka MM, Novotny PJ, Sloan JA, Kolars JC, Habermann TM, Shanafelt TD (2006). Association of perceived medical errors with resident distress and empathy: a prospective longitudinal study.. JAMA.

[3] Hojat M, Vergare MJ, Maxwell K, Brainard G, Herrine SK, Isenberg GA, Veloski J, Gonnella JS (2009). The Devil is in the Third Year: A Longitudinal Study of Erosion of Empathy in Medical School.. Academic Medicine.

[4] Park KH, Roh H, Suh DH, Hojat M (2014). Empathy in Korean medical students: Findings from a nationwide survey.. Med Teach.

[5] Bellini LM, Baime M, Shea JA (2002). Variation of mood and empathy during internship.. JAMA.

[6] Brazeau CMLR, Schroeder R, Rovi S, Boyd L (2010). Relationships Between Medical Student Burnout, Empathy, and Professionalism Climate.. Academic Medicine.

[7] Paro HB, Silveira PS, Perotta B, Gannam S, Enns SC, Giaxa RR, Bonito RF, Martins MA, Tempski PZ (2014). Empathy among medical students: is there a relation with quality of life and burnout?. PLoS ONE.

[8] Thomas MR, Dyrbye LN, Huntington JL, Lawson KL, Novotny PJ, Sloan JA, Shanafelt TD (2007). How do distress and well-being relate to medical student empathy? A multicenter study.. J Gen Intern Med.

[9] Dyrbye LN, Harper W, Durning SJ, Moutier C, Thomas MR, Massie FS, Eacker A, Power DV, Szydlo DW, Sloan JA, Shanafelt TD (2011). Patterns of distress in US medical students.. Med Teach.

[10] Newbury-Birch D, White M, Kamali F (2000). Factors influencing alcohol and illicit drug use amongst medical students.. Drug Alcohol Depend.

[11] Dahlin M, Joneborg N, Runeson B (2005). Stress and depression among medical students: a cross-sectional study.. Med Educ.

[12] Kjeldstadli K, Tyssen R, Finset A, Hem E, Gude T, Gronvold NT, Ekeberg O, Vaglum P (2006). Life satisfaction and resilience in medical school--a six-year longitudinal, nationwide and comparative study.. BMC Med Educ.

[13] Tempski P, Bellodi PL, Paro HB, Enns SC, Martins MA, Schraiber LB (2012). What do medical students think about their quality of life? A qualitative study.. BMC Med Educ.

[14] Haldorsen H, Bak NH, Dissing A, Petersson B (2014). Stress and symptoms of depression among medical students at the University of Copenhagen.. Scand J Public Health.

[15] Jeong Y, Kim JY, Ryu JS, Lee KE, Ha EH, Park H (2010). The Associations between Social Support, Health-Related Behaviors, Socioeconomic Status and Depression in Medical Students.. Epidemiol Health.

[16] Silva AG, Cerqueira ATAR, Lima MCP (2014). Social support and common mental disorder among medical students.. Rev bras epidemiol.

[17] Yamada Y, Klugar M, Ivanova K, Oborna I (2014). Psychological distress and academic self-perception among international medical students: the role of peer social support.. BMC Med Educ.

[18] Hojat M, Louis DZ, Maxwell K, Gonnella JS. The Jefferson Scale of Empathy (JSE): An update. Health Policy Newsletter. 2011;24(2):5-6.

[19] Roh MS, Hahm BJ, Lee DH, Suh DH (2010). Evaluation of empathy among Korean medical students: a cross-sectional study using the Korean Version of the Jefferson Scale of Physician Empathy.. Teach Learn Med.

[20] Cohen S, Kamarck T, Mermelstein R (1983). A global measure of perceived stress.. J Health Soc Behav.

[21] Lee OS. The relationship between the rehabilitation motive and social support perceived by spinal cord injury patients. Master's Program in of Social Welfare, Graduate School of Social Welfare, Seoul: The Catholic University of Korea; 2000.

[22] Dahlem NW, Zimet GD, Walker RR. The multidimensional scale of perceived social support: a confirmation study. J Clin Psychol. 1991;47(6):756-761. 10.1002/1097-4679(199111)47:6<756::aid-jclp2270470605>3.0.co;2-l 1757578

[23] Lee J, Shin C, Ko YH, Lim J, Joe SH, Kim S, et al. The reliability and validity studies of the Korean version of the Perceived Stress Scale. Korean J Psychosom Med. 2012;20(2):127-34.

[24] Wang X, Cai L, Qian J, Peng J (2014). Social support moderates stress effects on depression.. Int J Ment Health Syst.

[25] Voltmer E, Kieschke U, Schwappach DL, Wirsching M, Spahn C (2008). Psychosocial health risk factors and resources of medical students and physicians: a cross-sectional study.. BMC Med Educ.

[26] Shanafelt TD, West C, Zhao X, Novotny P, Kolars J, Habermann T, Sloan J (2005). Relationship between increased personal well-being and enhanced empathy among internal medicine residents.. J Gen Intern Med.

[27] Wolf TM, Scurria PL, Webster MG (1998). A Four-year Study of Anxiety, Depression, Loneliness, Social Support, and Perceived Mistreatment in Medical Students.. J Health Psychol.

[28] Fahrenkopf AM, Sectish TC, Barger LK, Sharek PJ, Lewin D, Chiang VW, Edwards S, Wiedermann BL, Landrigan CP (2008). Rates of medication errors among depressed and burnt out residents: prospective cohort study.. BMJ.

[29] Shanafelt TD, Bradley KA, Wipf JE, Back AL. Burnout and self-reported patient care in an internal medicine residency program. Ann Intern Med. 2002;136(5):358-67. 10.7326/0003-4819-136-5-200203050-00008 11874308

[30] Williams ES, Manwell LB, Konrad TR, Linzer M (2007). The relationship of organizational culture, stress, satisfaction, and burnout with physician-reported error and suboptimal patient care: results from the MEMO study.. Health Care Manage Rev.

[31] Grühn D, Rebucal K, Diehl M, Lumley M, Labouvie-Vief G (2008). Empathy across the adult lifespan: Longitudinal and experience-sampling findings.. Emotion.

[32] Kiessling C, Schubert B, Scheffner D, Burger W (2004). First year medical students' perceptions of stress and support: a comparison between reformed and traditional track curricula.. Med Educ.

